# Effects of a commercial whitening toothpaste containing hydrogen peroxide and citric acid on dentin abrasion and erosion

**DOI:** 10.1186/s12903-023-03319-x

**Published:** 2023-09-01

**Authors:** Jae-Heon Kim, Soyeon Kim, Young-Seok Park

**Affiliations:** 1 Department of Oral Anatomy and Dental Research Institute, School of Dentistry, Seoul National University, 101 Daehak-ro, Jongno-gu, Seoul, 03080 Republic of Korea; 2 Center for Future Dentistry, School of Dentistry, Seoul National University, Seoul, 03080 Republic of Korea

**Keywords:** Whitening toothpaste, Hydrogen peroxide, Citric acid, Dentin abrasion, Dentin erosion

## Abstract

**Background:**

Hydrogen peroxide (HP) and citric acid (CA), key contributors to toothpaste acidity, can lead to dental loss. This study aimed to compare the amount of abrasion or loss of dentin based on pH, buffering, and concentration of HP and CA in commercial and experimental toothpastes after toothbrushing or immersion.

**Methods:**

Bovine dentin specimens were randomly assigned to nine solutions. The prepared solutions included two commercial toothpastes (whitening toothpaste [WT] with HP and CA; conventional toothpaste [CT] without HP and CA), reference slurry (RS), two CA solutions (1.92%, CAS1; 0.001%, CAS2), basic solution (7.16% sodium phosphate dibasic [SPDS]), CA phosphate buffer solution (3.58% SPDS and 0.96% CA [CAPB]), HP solution (4%, HPS), and distilled water (DW). Dentin specimens were performed in two treatments: one with only abrasion (10,000 brushings) and one with only immersion (1 h). After treatments, the amount of dentin loss and surface images were measured and observed using noncontact profilometry. Data were analyzed using an one-way analysis of variance and the Tukey test as a post hoc analysis (*p* < 0.05).

**Results:**

WT with pH 5.0 had lower dentin abrasion than CT and RS after brushing but had higher dentin loss than both after immersion. The dentin surfaces of CAS1, CAPB, and WT were damaged after immersion, whereas HPS, CAS2, CT, SPDS, RS, and DW remained intact after soaking. CAS2 and HPS, which had a pH of 5.0 like WT, did not significantly differ from those of DW after brushing.

**Conclusions:**

WT containing HP and CA did not cause significant dentin abrasion but may cause additional dentin loss even without brushing. After brushing or immersion, the CA concentration may affect the dentin surface more than the HP concentration included in WT. The amount of abrasion or loss of dentin after brushing or soaking can vary based on the composition, concentration, and buffer in the solution, even if the pH of the solution is similar to pH 5.0.

## Background

Consumers can now simply whiten their teeth at home using whitening toothpaste (WT), a popular product, to improve their smiles. WTs typically contain higher abrasive content and various types of abrasives and chemicals, such as hydrogen peroxide (HP) and optical brightening agents (e.g., blue covarine) [1]. WTs can be applied directly to the tooth surface using a single or combined strategy. It can remove plaque and stains on the tooth exterior or improve discoloration on the tooth interior when one brushes. Consequently, WTs can help improve aesthetics by giving the appearance of brighter teeth than before [2].

HP is a chemical component commonly included as an active ingredient in WTs among other additives. Although WTs have various HP concentrations depending on brands and products, the regulations regarding the maximum allowable HP concentration in WT may vary from country to country [3]. For example, the concentration of HP in WTs containing HP that consumers can buy without a prescription is ≤ 0.1% in the European Union and ≤ 3% in South Korea [4]. Due to its low molecular weight, HP can act directly on porous enamel and dentin and can also decompose into water and oxygen via an oxidation–reduction reaction. The free radicals produced during HP decomposition can break the double bonds of coloring molecules. In other words, they reduce the number of coloring molecules that absorb light, thereby increasing tooth-light reflectance. Therefore, teeth can appear brighter with improved whitening [3].

The pH of WTs containing HP is generally below 5, which is significantly lower than that of conventional toothpastes and WTs without HP [5, 6]. Low pH can adversely affect the surface of dental hard tissues such as enamel and dentin by potentially causing erosion. Tooth erosion is the chemical dissolution of dental hard tissues by nonbacterial acids [7]. It may cause irreversible damage to these tissues, increased sensitivity, a greater risk of tooth fracture, and esthetic deterioration [8].

WTs containing HP may have different pH levels depending on HP concentration. Notably, additives other than HP may also affect pH by adjusting it. WTs containing HP often include citric acid (CA) as a stabilizer. CA, which can adjust the pH of various products, is a commonly included ingredient in beverages, foods, pharmaceuticals, and dental products [9]. However, interactions between CA and dental hard tissues can lead to surface demineralization and probably increased susceptibility to dental abrasion [10, 11]. Dentin is more susceptible to abrasion and erosion than enamel due to compositional differences and low modulus of elasticity. It can cause more extensive loss than enamel [12].

The commercial WTs incorporating HP have become prevalent more and more to satisfy consumer demand for at-home tooth whitening. They can be used regularly to achieve whiter teeth. In a previous study, WTs containing HP demonstrated a lower relative dentin abrasion–profilometry equivalent (RDA–PE) than conventional toothpastes and WTs containing sodium bicarbonate, although WT with HP had a subacid pH compared to other toothpastes [5] which was likely to cause erosion and was a condition that may make it vulnerable to abrasion [13–15].

HP and CA are key factors that contribute to toothpaste acidity and can lead to eroded surface [10, 16]. Although numerous factors can affect the amount and feature of tooth surface abrasion or loss after brushing or immersion with WTs, studies regarding the effects of pH and acidic constituents of WTs, as well as their interaction, remain scarce. Therefore, this study compares the amount of abrasion or the loss of dentin surfaces resulting from the use of commercial and experimental toothpastes, considering key factors such as pH, buffering, and HP and CA concentrations, after toothbrushing or immersion.

## Materials and methods

### Bovine dentin specimens

**Fig. 1:**
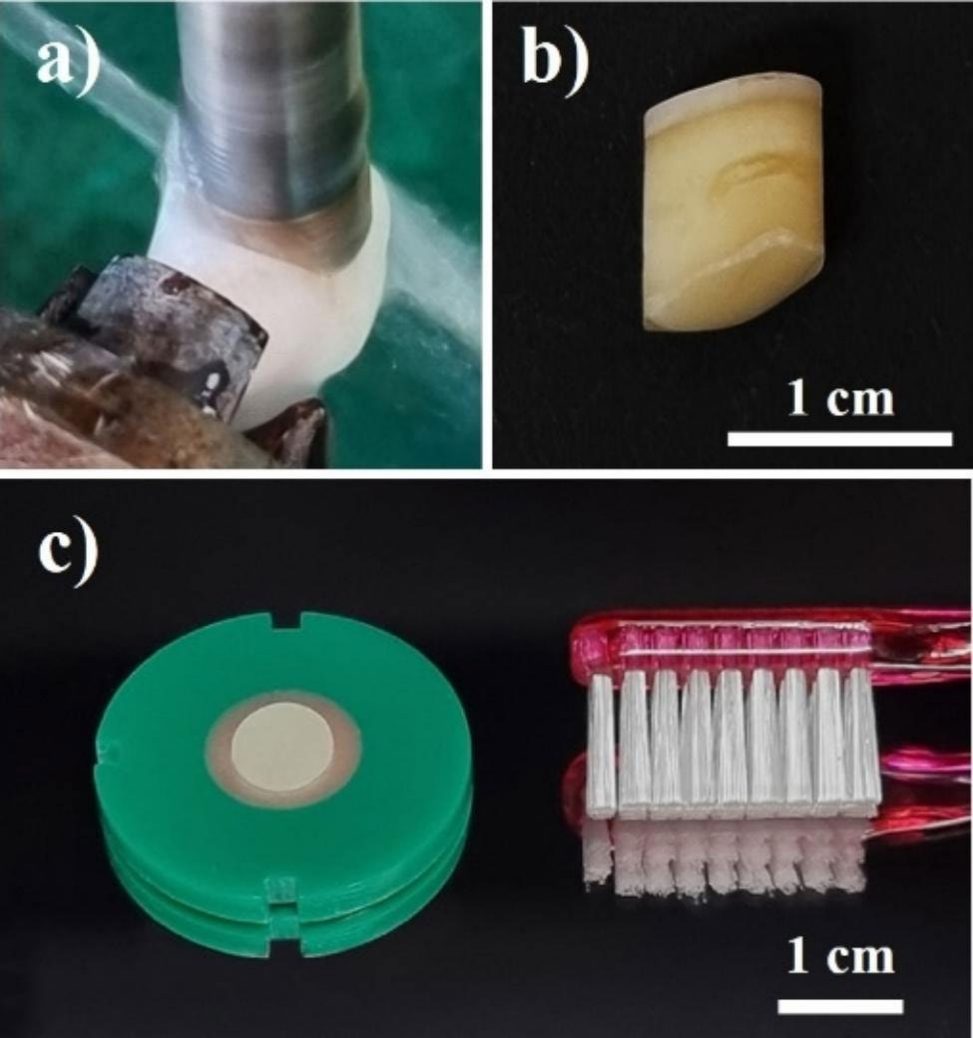
(**a**) Perforation of a tooth extracted while spraying with water. (**b**) Perforated tooth with a diameter of 8 mm. The top is the labial surface, whereas the bottom is the lingual surface. (**c**) A completed bovine dentin specimen and three rows of bristles were used for the experiment

A total of 144 bovine dentin specimens were prepared for the experiment. Incisors without defects such as caries or fractures were extracted from the mandibles of cows. The central part of the crown was drilled from the labial surface to the lingual surface so that the dentin tubules and the direction of toothbrushing were perpendicular using a perforator (punched diameter, 8 mm; YDM-13 mm, Yongsoo Precision, Daegu, Korea) while spraying water (Fig. 1a) [17, 18]. Extracted teeth with a dentin thickness of at least 2 mm were selected to prepare the dentin specimens (Fig. 1b). The teeth were placed in an acrylic ring with outer and inner diameters of 30 and 12 mm, respectively, and the teeth were fixed by pouring self-curing resin (Vertex Self-Curing, Vertex, Zeist, Netherlands). The resin hardened for one week. The dentin specimens were stored in a container maintained at 100% relative humidity while the self-curing resin was sufficiently cured. The specimen surfaces were polished flat using a polishing machine (KDPI-330, KD Precision, Bucheon, Korea) and sandpaper (#220, 600, 1200, and 2000; R&B, Daejeon, Korea). Dentin specimens did not separately remove the dentin smear layer during the specimen preparation because the removal of the smear layer requires exposure to acid, which may affect the dentin surface [19]. A load of 300 g was applied to the specimen surface using a Vickers hardness tester (HMV-2, Shimadzu, Tokyo, Japan). An average value was calculated by measuring five points per specimen. Specimens corresponding to the values of 30–70, which is the Vickers hardness range for dentin, were obtained. Finally, concave specimens with an average depth of the measured surface exceeding 0.3 μm were excluded before the experiment using noncontact surface profilometry with a detection limit of 270 μm (NV-1800, NanoSystem, Daejeon, Korea) (Fig. 1c).

### Preparation of experimental solutions

Two commercial toothpastes, which consists of one WT containing HP and CA and one conventional toothpaste (CT) without HP and CA, were prepared. Both commercial toothpastes were vigorously mixed in distilled water (DW) at a ratio of 1:1.6 (25 g / 40 ml) according to the International Organization for Standardization (ISO) 11609 [5]. To prepare a reference slurry (RS), a reference diluent solution containing 10% glycerin (99.5%, Shanghai Aladdin Biochemical Technology, Shanghai, China), 0.5% carboxymethyl cellulose (CMC, Sigma-Aldrich, St., Louis, USA) and 89.5% DW was prepared. RS was prepared by mixing calcium pyrophosphate (99.95%, Strem Chemicals, Newburyport, USA) as a standard reference abrasive and the reference diluent solution at a ratio of 1:5 (10 g/50 ml) according to ISO 11609 [5]. CA solutions were prepared by dissolving or diluting CA (251275, Sigma-Aldrich, Steinheim, Germany) in DW to prepare 1.92% (CAS1) and 0.001% (CAS2) CA solutions. A CA phosphate buffer (CAPB) was prepared by mixing 7.16% sodium phosphate dibasic solution (SPDS; Junsei Chemical, Tokyo, Japan), which was not containing abrasive, and 1.92% CA solution to a pH level of 5.0 [20]. The HP solution (HPS) was prepared by diluting 35% HP (18304, Sigma-Aldrich, St. Louis, USA) at a concentration of 4%, and the HPS concentration was checked using a digital refractometer (PR-50HO, ATAGO, Tokyo, Japan) with ± 0.5% accuracy. Lastly, DW was prepared.

The experimental solutions were measured using a pH meter (F-71, HORIBA, Kyoto, Japan) and pH electrode (9615 S-10D, HORIBA) calibrated with three pH buffer kits (pH 4.0, 7.0, and 10.0, 502-S, HORIBA, Singapore). The temperature was 25℃ ± 0.5℃ when the pH was measured. Table 1 lists the ingredients, concentrations, and pH of the toothpastes and solutions used in the experiment.

**Table 1:** Detailed ingredients, concentrations, and pH of the toothpaste and solution used in the experiment

Codes †	Productname /Manufacturer	Ingredients	pH	Fluoride (ppm)	Lot
WT	Vussen 28 ^a^	Hydrogen peroxide 35% (containing 2.8%), Colloidal silicon dioxide, Glycerin, Sodium lauryl sulfate, Sodium metaphosphate, Sodium saccharin, Citric acid, L-Menthol, Purified water, Poloxamer 407, Polyethylene glycol 1500, Flavor, Hydroxyethylcellulose	5.011 ± 0.038	━	22H007
CT	Perioe new fresh alpha ^b^	Sodium monofluorophosphate, Calcium carbonate, Glycerin, Sodium lauryl sulfate, Polyoxyethylene sorbitan monooleate, Amorphous sorbitol solution 70%, Saccharin sodium hydrate, Disodium dihydrogen pyrophosphate, Zinc acetate, Xylitol, Purified water, Carboxymethylcellulose sodium salt, Silica (TIXOSIL 43 K), Flavor	7.774 ± 0.020	1000	FB23C
RS	Reference slurry	Calcium pyrophosphate, 10% glycerin, 0.5% carboxymethyl cellulose	6.850 ± 0.019	━	━
CAS1	Citric acid solution 1	1.92% citric acid	2.026 ± 0.009	━	━
CAS2	Citric acid solution 2	0.001% citric acid	5.001 ± 0.030	━	━
CAPB	Citric acid phosphate buffer	3.58% sodium phosphate dibasic and 0.96% citric acid	5.006 ± 0.003	━	━
SPDS	Sodium phosphate dibasic solution	7.16% sodium phosphate dibasic	9.077 ± 0.008	━	━
HPS	Hydrogen peroxide solution	4% hydrogen peroxide	5.022 ± 0.018	━	━
DW	Distilled water	━	6.704 ± 0.054	━	━

^†^ WT = Whitening toothpaste, CT = Conventional toothpaste, RS = Reference slurry, CAS = Citric acid solution, CAPB = Citric acid phosphate buffer, SPDS = Sodium phosphate dibasic solution, HPS = Hydrogen peroxide solution DW = Distilled water

^a^ Osstem pharma, Ansan, Korea, ^b^ LG Household & Health Care, Cheongju, Korea

### Dentin abrasion and immersion process

For the abrasion test of the dentin surface, a window with 4 × 20 mm (width × height) was created using a 25 μm polyester tape (162.H421.25B, HaeSung Tape, Daejeon, Korea) to produce a reference surface that was not brushed on the specimen surface before testing. Each solution was poured after fixing eight specimens in the bath (n = 8). Toothbrushing was performed 10,000 times [5, 21] at a speed of 170 strokes per minute using an automatic brushing machine (RB118, R&B, Daejeon, Korea) and a three-row flat-bristle toothbrush (Name Brush T21, Guardian Angel, Suwon-si, Korea) with a bristle’s diameter of 178 μm (Fig. 1c). The load applied to the toothbrushes was 150 g.

In the immersion test, a window of the same size as the brushing process was created, and eight specimens were assigned to each solution (n = 8). Specimens were immersed for 1 h, equivalent to 10,000 brushing times with stirring at 300 rpm.

After the abrasion and immersion tests, the tape was removed from each specimen and washed thoroughly with tap water.

### Dentin surface measurement by noncontact profilometry and RDA-PE calculation

After brushing and immersion, the specimens were measured in continuous mode with a size of 2.304 × 1.728 mm (width × height) per measurement area from the left reference surface to the right reference surface at 5x magnification using a noncontact surface profilometry. The analysis size was 1 mm on each reference surface and 4 mm exposed to brushing or immersion. The total width was 6 mm, and the height was 1.5 mm. Based on both reference planes, the average depth of the lost dentin by brushing or immersion was calculated using analysis software (NanoMap Ver. 3.5.17.7).

The RDA–PE value, which is the relative abrasion value of dentin, was determined using the following formula: the average depth of dentin brushed with each solution divided by the average depth of dentin brushed with RS multiplied by 100. In this study, 10,000 strokes of RS corresponded to RDA–PE 100 [5].

### Observation, size, and content of abrasive

To observe the abrasives of CT, RS, and WT, which contained abrasives among the nine solutions, 1 g of commercial toothpastes (CT and WT) was added to 50 ml of DW and vigorously stirred to dissolve. The mixed solutions were centrifuged at 10,000 RPM for 15 min using a centrifuge (Avanti J-E, Beckman Coulter, Fullerton, USA) to separate the abrasive. The supernatant in the tube was discarded, and this process was repeated five times. Finally, the abrasives were rewashed with ethanol. The washed abrasives were completely dried at 37 °C for three days using a drying oven (DO-49, Daeheung, Incheon, Korea). RS did not undergo the cleaning process and used directly calcium pyrophosphate powder (reagent). The abrasives were fixed to carbon tape on stubs and coated with platinum. They were observed at 3,000x magnification using a field emission scanning electron microscope (FE-SEM; Apreo S LoVac, Thermo fisher scientific, MA, USA). The abrasives were analyzed for elements in the visualized area using energy-dispersive X-ray spectroscopy (EDS; XFlash 6160, Bruker, Berlin, Germany) and analysis software (ESPRIT, ver. 2.1, Bruker).

An ultrasonic cleaner was used to disperse 30 mg of dried abrasives from CT, WT, and RS into DW for a size study of abrasives. The average particle size of each abrasive was analyzed in triplicate using a particle size analyzer (LA-950V2, HORIBA, Kyoto, Japan) (n = 3).

To determine the abrasive content of the CT, WT, and RS, 1 g of each solution was deposited in an alumina crucible. The crucible was covered with a lid and weighed using an electronic balance (HS220S, Hansung, Hwaseong, Korea). It was placed in an electric furnace (NEY 6-1350 A, NEY, CA, USA) and heated at 2 °C per minute to 600 °C to incinerate the residue. The incinerator temperature was maintained at 600 °C for 2 h and cooled slowly. After incineration, the changed weight of the crucible was measured, and the included abrasives were calculated as a weight% (wt%). The wt% of the abrasive was measured in triplicate each (n = 3).

### Statistics

The sample size for each of the 9 solutions in 2 treatments, which were toothbrushing and immersion, was calculated using G*Power software (Ver.3.1.9.6) according to the pilot study. It was considered 80% power and a 5% significance level and eight specimens were required per group. Each loss data on the dentin surface due to brushing or immersion was analyzed using statistics software (SPSS statistics v26.0, IBM, NY, USA). Before analysis, the normality test was checked using the Kolmogorov–Smirnov test. Data were analyzed using a one-way analysis of variance (ANOVA), followed by a Tukey test as a post-hoc test. The level of significance was 5%.

## Results

### Dentin abrasion by toothbrushing

Table 2 lists the mean and standard deviation of the RDA–PE values and the average depth of the abrasion on the dentin surface. A significant difference was observed between groups based on the RDA–PE value and the average depth of abrasion (p < 0.001), with CAS1 presenting the highest values, while no significant difference was observed regarding CT. CAS1 and CT were significantly different from other groups (p < 0.05). Each of RS, CAPB, and WT showed significant differences in RDA–PE value and the average depth of abrasion from other groups (p < 0.05). DW had the lowest RDA–PE value and average depth of abrasion; CAS1, CT, RS, CAPB, and WT differed significantly (p < 0.05), and HPS, CAS2, and SPDS did not.

**Table 2:** Means and standard deviation of the RDA–PE and average depth of dentin by toothbrushing

Solution	RDA-PE	Average depth of abrasion (µm)
CAS1 (pH 2.0)	162.0 ± 30.0 ^a^	55.61 ± 10.14 ^a^
CT (pH 7.8)	155.0 ± 22.0 ^a^	53.22 ± 7.45 ^a^
RS (pH 6.9)	100.0 ± 29.0 ^b^	34.29 ± 10.11 ^b^
CAPB (pH 5.0)	62.0 ± 8.0 ^c^	21.24 ± 2.66 ^c^
WT (pH 5.0)	29.0 ± 5.0 ^d^	10.10 ± 1.71 ^d^
HPS (pH 5.0)	3.0 ± 0.4 ^e^	1.10 ± 0.15 ^e^
CAS2 (pH 5.0)	3.0 ± 0.7 ^e^	0.95 ± 0.24 ^e^
SPDS (pH 9.1)	1.0 ± 0.5 ^e^	0.41 ± 0.19 ^e^
DW (pH 6.7)	1.0 ± 0.3 ^e^	0.35 ± 0.12 ^e^

The RDA-PE values and average depth of abrasion was described in descending order

Different letters imply significant differences between groups (p < 0.05; n = 8)

**Fig. 2:**
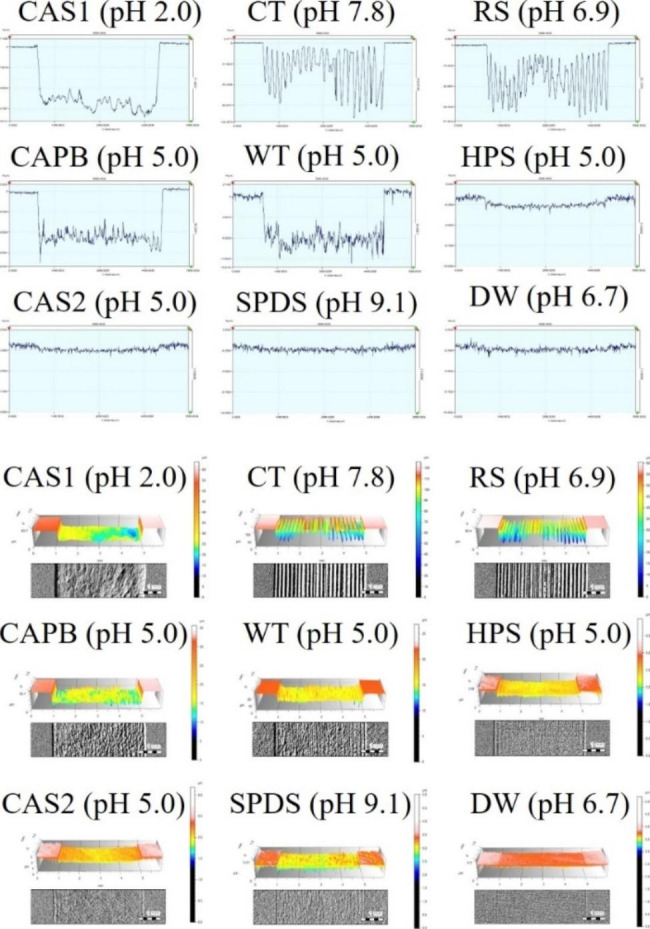
Two-dimensional (2D), three-dimensional (3D), and optical images of the abrasion surface on the dentin after 10,000 brushings in each solution. The scale bar size is 1 mm in the optical images. Code: CAS1 (1.92% citric acid solution); CT (Conventional toothpaste; Perioe new fresh alpha); RS (Reference slurry); CAPB (3.58% sodium phosphate dibasic and 0.96% citric acid; Citric acid phosphate buffer); WT (Whitening toothpaste; Vussen 28); HPS (4% hydrogen peroxide solution); CAS2 (0.001% citric acid solution); SPDS (7.16% Sodium phosphate dibasic solution); DW (Distilled water)

Figure 2 shows the two-dimensional (2D), three-dimensional (3D), and optical images on the dentin surface based on the solution type after toothbrushing. Dentin surface of HPS, CAS2, SPDS, and DW observed minor abrasion compared to CAS1, CT, RS, CAPB, and WT.

### Dentin loss by immersion

Table 3 lists the mean and standard deviation of the average depth of the dentin loss after immersion for 1 h in each solution. The mean depth values significantly differed between groups (p < 0.001). CAS1 had the highest depth value, and the other groups significantly differed (p < 0.05). CAPB significantly differed from all groups, excluding WT. DW had the lowest depth value, CAS1, CAPB, and WT groups differed significantly (p < 0.05), and HPS, CAS2, CT, SPDS, and RS did not.

**Table 3:** Means and standard deviation of the average depth of dentin loss by immersion

Solution	Average depth of dentin (µm)
CAS1 (pH 2.0)	23.44 ± 3.29 ^a^
CAPB (pH 5.0)	3.21 ± 0.91 ^b^
WT (pH 5.0)	2.57 ± 0.65 ^b^
HPS (pH 5.0)	0.38 ± 0.11 ^c^
CAS2 (pH 5.0)	0.33 ± 0.13 ^c^
CT (pH 7.8)	0.30 ± 0.05 ^c^
SPDS (pH 9.1)	0.30 ± 0.07 ^c^
RS (pH 6.9)	0.27 ± 0.07 ^c^
DW (pH 6.7)	0.26 ± 0.06 ^c^

The average depth of dentin loss was described in descending order

Different letters imply significant differences between groups (p < 0.05; n = 8)

**Fig. 3:**
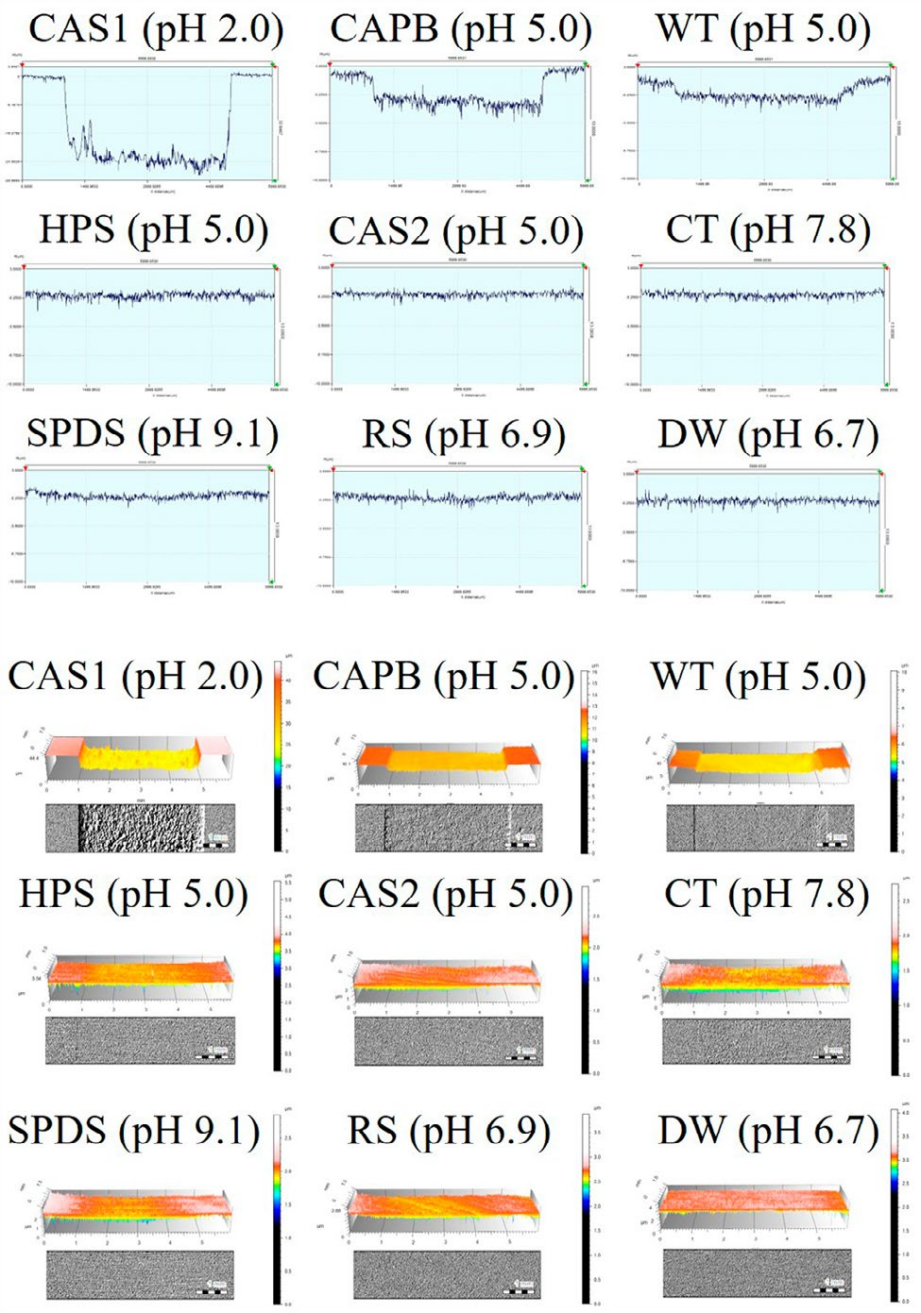
The 2D, 3D, and optical images of the dentin surface after immersion for 1 h in each solution. The scale bar size is 1 mm in the optical images. Code: CAS1 (1.92% citric acid solution); CAPB (3.58% sodium phosphate dibasic and 0.96% citric acid; Citric acid phosphate buffer); WT (Whitening toothpaste; Vussen 28); HPS (4% hydrogen peroxide solution); CAS2 (0.001% citric acid solution); CT (Conventional toothpaste; Perioe new fresh alpha); SPDS (7.16% Sodium phosphate dibasic solution); RS (Reference slurry); DW (Distilled water)

Figure 3 shows the 2D, 3D, and optical images on the dentin surface after immersion in the solutions. Surface damage and changes were detected on the dentin surface in contact with CAS1, CAPB, and WT compared to the reference surface. HPS, CAS2, CT, SPDS, and RS showed dentin surfaces similar to DW, and almost no loss of dentin occurred when comparing the reference and exposed surfaces.

### Observation, size, and content of abrasive

**Fig. 4:**
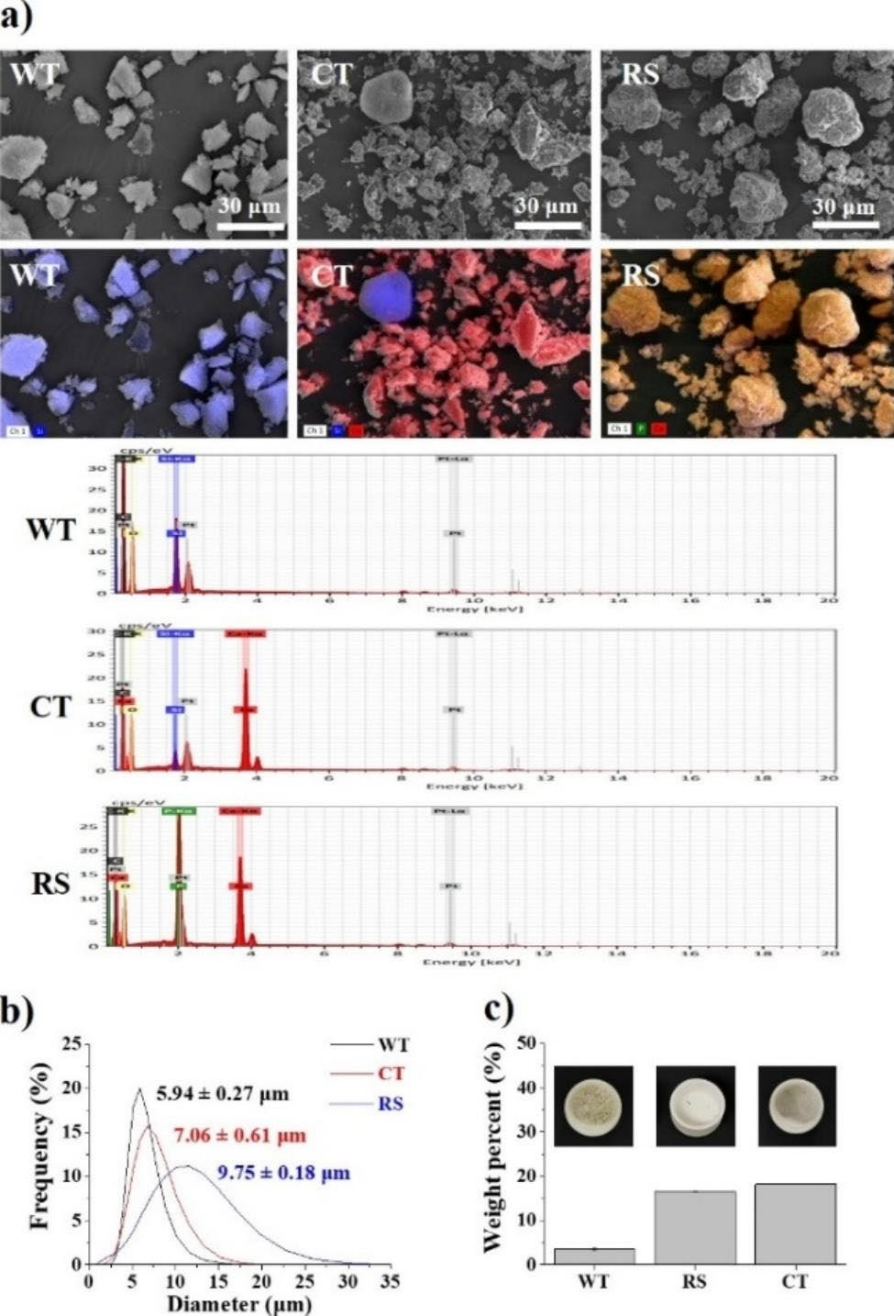
(**a**) Observation and element analysis of abrasives using field emission scanning electron microscopy (FE–SEM) and energy-dispersive X-ray spectroscopy (EDS). Images of all abrasives were obtained at the same magnification of 3,000x and the size of the white scale bar is 30 μm. The main elements of the abrasives by EDS analysis are indicated in the lower left corner of the EDS mapping image, and all components detected are shown in the spectrum image. The analyzed abrasive is matched to the FE–SEM image with the corresponding elemental color. (**b**) Distribution of average particle size of abrasives in WT, CT, and RS. (**c**) Weight% (wt%) of abrasive in WT, RS, and CT solutions. Photographs of the abrasive remaining in the crucible after incineration of the WT, RS, and CT solutions at 600 °C in a furnace. The wt% of abrasive in WT, RS, and CT was 3.57 ± 0.23%, 16.60 ± 0.10%, and 18.27 ± 0.06%, respectively in the graph. Code: WT (Whitening toothpaste; Vussen 28); CT (Conventional toothpaste; Perioe new fresh alpha); RS (Reference slurry)

The abrasives of WT, CT, and RS were observed in various sizes and had polygonal or circular shapes, as shown in the FE–SEM images (Fig. 4a). Most of the abrasives were observed within 30 μm in all three groups. The abrasives in WT had relatively smaller abrasive than CT and RS. The elements of the abrasives were analyzed in the observed area by EDS, and the abrasives corresponding to each were matched (mapping) to FE–SEM images (Fig. 4a). Silica (blue color) in WT, silica and calcium (red color) in CT, and calcium and phosphorus (green color) in RS were mainly detected.

As determined by the analysis of particle size distribution, the average abrasive sizes of WT (5.94 ± 0.27 μm) were smaller and more homogeneous than those of CT (7.06 ± 0.61 μm) and RS (9.75 ± 0.18 μm) and were similar to those visualized by FE–SEM (Fig. 4b).

After incineration at 600 °C in the furnace, the wt% of abrasive in WT, RS, and CT was 3.57 ± 0.23%, 16.60 ± 0.10%, and 18.27 ± 0.06%, respectively (Fig. 4c).

## Discussion

Using noncontact surface profilometry, the amount of dentin abrasion or dentin loss was measured by RDA-PE after 10,000 brushings or immersion for 1 h and was compared among various solutions. Among them, CAS2, CAPB, and HPS with pH of 5.0 have the similar level of pH to WT containing HP and CA. The effect of solutions with similar pH of 5.0 on dentin loss was compared, although they have different ingredients or concentrations. RS was included to obtain the RDA-PE value, and CT with non-acidic pH was involved in evaluating the amount of dentin abrasion and erosion. In addition, CAS1 and SPDS were assessed because they are used to prepare CAPB, which has higher concentration of CA than CAS2, even if it has the same level of pH with CAS2. Finally, DW was used as a negative control. The pH of WT (5.011 ± 0.038), which was below 6.0, the critical pH of dentin [22, 23], was sub-acidic in contrast to CT, RS, SPDS, and DW in the present study (Table 1).

A solution with a relatively low pH may cause demineralization and loss of dentin by erosion [24], although it may be advantageous for preventing HP decomposition [25]. According to a previous study, HP oxidation was mitigated when CA was added to a 0.3% HP solution adjusted to a pH range of 2–6 rather than an alkali solution of pH 8.0 or higher under harsh conditions of 40℃ for six weeks [26]. CA can lower the pH of HP solutions and may be used as a stabilizer due to the slow decomposition of HP. Low pH can act as a preservative by preventing bacterial growth [27]. In other words, adding CA to WTs containing HP can be beneficial for maintaining HP concentration and long-term storage of dentifrice. CA can directly dissolve tartar, which can help HP to immediately act on teeth, functioning as an accelerator for tooth whitening [28]. Compared to other acids, CA is readily available, has a low pKa, and is inexpensive [26].

Solutions with a low pH can be pH modified or buffered with various additives to reduce tooth loss [29, 30]. In the current study, CAPB was mixed with CAS1 (pH 2.026 ± 0.009) and SPDS (pH 9.077 ± 0.008) to achieve a pH of 5.0 similar to WT; as a result, the pH of CAPB was corrected and buffered (Table 1). For all solutions, the amount of abrasion on the dentin after brushing was determined by the RDA–PE value.

The RDA–PE value, which is the relative abrasion value of dentin by the profilometry method, can quantify the degree of dentin abrasion compared with the RS based on ISO 11609. Profilometry is a well-established method for evaluating dentin abrasion or erosion [30]. Noncontact profilometry can observe by 2D, 3D, and optical imaging of the overall dentin surface without damaging the exposure and reference surfaces [4]. ISO 11609 considers the RDA–PE value of RS to be 100 and limits the maximum RDA–PE value to 250 [5].

A low pH can soften the dentin surface, causing more abrasion [31]. Among all groups, CAS1 had the lowest pH (2.026 ± 0.009), and the RDA–PE value (162.0 ± 30.0) was the highest after toothbrushing, although an abrasive was not included in the solution (Tables 1 and 2). As the pH of CAPB was buffered and adjusted to the 5.0 level, the CA concentration in the solution was approximately half (50%) the difference between CAS1 and CAPB, and the RDA–PE value of CAPB (62.0 ± 8.0) was reduced by more than half (about 62%) compared to that of CAS1. The size of the abrasion in CAPB also decreased compared to CAS1. Interestingly, despite the lack of abrasives, CAPB had a higher RDA–PE value than WT containing abrasives (29.0 ± 5.0); its RDA–PE value significantly exceeded those of CAS2 (3.0 ± 0.7) and HPS (3.0 ± 0.4) that lacked abrasives and had a pH of 5.0. These findings suggest that, even at equal pH levels of 5.0, RDA–PE values can differ depending on the CA concentration or other ingredients present in the solution.

The amount of dentin abrasion may be closely related to factors such as the type, content, characteristics (e.g., size, shape, hardness, and homogeneity) of the abrasive, other additives, acid, and pH in the solution during brushing [32–36]. In this study, WT had a significantly lower RDA–PE value (29.0 ± 5.0) than RS (100.0 ± 29.0) and CT (155.0 ± 22.0). WTs may raise concerns regarding increased abrasion on the dentin surface because they are often conjectured to contain more abrasives than conventional toothpastes [37–39]. However, this study showed that WT containing HP (3.57 ± 0.23%) had significantly lower abrasive content than RS (16.60 ± 0.10%) and CT (18.27 ± 0.06%). Although the type, size, and content of the abrasive in this study may have varied (Fig. 4), a complex mechanism of factors including the properties of the abrasive, other ingredients containing CA, and low pH during toothbrushing may have induced differences in RDA–PE values among WT, CT, and RS.

After immersion in the solutions, CAS1 (23.44 ± 3.29 μm) with pH 2.0 caused 90 times more dentin loss than DW (0.26 ± 0.06 μm) without brushing, and damage was also found in the surface of dentin in contact with the solution (Table 3; Fig. 3). CAPB (3.21 ± 0.91 μm) buffered to pH 5.0 had its value reduced by approximately 86% compared to CAS1. WT (2.57 ± 0.65 μm) containing HP and CA caused approximately 10 times more dentin loss than DW, and the dentin surface exposed to the solution was similar to CAS1 and CAPB (Fig. 3). Here, note that HPS (0.38 ± 0.11 μm), which contained a 4% concentration of HP higher than the 2.8% concentration of HP included in the WT, CAS2 (0.33 ± 0.13 μm), which contained a relatively low CA concentration, and the solutions had a pH of 5.0. However, neither solution induced significant dentin loss and surface changes compared to DW, indicating that factors such as CA concentration may have contributed more to the damage on the dentin surface than the low pH itself that the HP concentration causes in this case. Although this study focused on the amount of dentin loss after immersion in the solution, the collagen layer of the dentin surface may be changed that cannot be measured by noncontact profilometry [19]. Therefore, additional studies need to be accompanied by observation of the microstructure on the dentin surface. During data exploration, 2-way ANOVA was tried with brushing and immersion as one explanatory variable and solutions as another explanatory variable, and statistical significance was confirmed according to the two explanatory variables. However, the difference shown in brushing and immersion was predictable prior to the experiment, the comparisons within each group were analyzed statistically.

pH is usually a measure of the relative amounts of free hydrogen and hydroxyl ions and can be perceived as relative acidity in a solution [40]. However, pH trends may not be similar to acidity trends and may not be synonymous [41, 42]. Loss of dentin surface may vary depending on factors such as acid properties (e.g., type, concentration, and chelating effect), pH, buffering capacity, or amount of titrate acid [29, 30, 43]. Previous studies have demonstrated that a 6% CA solution was more effective than a 3% HP solution in removing the smear layer of dentin [44]. The solutions of three CA concentrations (0.07%, 0.25%, and 1.00%) with a similar pH range of 3.60–3.77 buffered with sodium citrate caused more dentin loss with increasing CA concentration [45]. In addition, the effect of removing dentin smears for 1 min was not significantly different between solutions with a pH of 2.0 or less plus a high concentration of 25% or 50% CA and the pH 6.0 level group buffered with sodium hydroxide. In other words, a high CA concentration in solutions could cause significant dentin surface loss even if the pH was buffered [46]. These results corroborate our findings [44–46].

Because commercial WTs have lower HP concentrations than professional whitening treatments, teeth may require long-term brushing in order to achieve the desired whitening effect [47]. In the present study, 10,000 brushings could clinically be equivalent to approximately one year of cumulative brushing per tooth [48]. Therefore, the amount of accumulated dentin abrasion or erosion after brushing or immersion following long-term use of WT containing HP and CA was evaluated. In addition, 10,000 brushings may be advantageous in measuring the amount of dentin abrasion in the solutions with low abrasivity [5]. Even though the remineralization process and intermittent brushing in real life are not taken into consideration in the present study, long-term use of WT containing HP and high CA concentrations may cause additional dentin damage other than brushing, with or without pH buffering. Information about the acid used in commercial WT may not be provided due to trade secrets in the packaging if not compulsory [49].

WT did not contain fluoride as a component compared to CT in this study (Table 1). Fluoride is an important ingredient present in toothpastes to prevent tooth caries and erosion [50] and to increase tooth remineralization [51]. Previous studies have reported that using toothpaste with fluoride may have benefits in preventing tooth caries [52] and can reduce tooth abrasion and erosion [53, 54]. However, adding of fluoride to WTs may cause interference between ions and reduce the diffusion of HP [55, 56]. Because of these concerns, manufacturers may be reluctant to include fluoride in WTs containing HP [4, 57]. This study focused on the effects of HP and CA contained in WT on the dentin surface without being affected by fluoride. However, WTs with HP may contain different types of acids [58], various concentrations, and fluoride, and the pH of WT can be buffered with other additives, which may have different effects on dentin abrasion and erosion, necessitating further study.

## Conclusion

In this study, commercial WT containing HP and CA with weak acidity did not cause significant dentin abrasion than RS and CT after toothbrushing. However, it may cause further dentin erosion than RS and CT after immersion. CA concentration is a factor that can have more influence on dentin abrasion, dentin erosion, and dentin surface than HP concentration contained in WT. The amount of dentin abrasion or dentin erosion can vary significantly depending on the composition, concentration, and buffer, even if the pH of the solution is similar to pH 5.0.

## Data Availability

All data generated or analysed during this study are included in this published article [and its supplementary information files].

## List of abbreviations

WT: Whitening toothpaste
HP: Hydrogen peroxide
CA: Citric acid
RDA-PE: Relative dentin abrasion–profilometry equivalent
CT: Conventional toothpaste
DW: Distilled water
RS: Reference slurry
CAS1: Citric acid solution1
CAS2: Citric acid solution2
CAPB: Citric acid phosphate buffer
SPDS: Sodium phosphate dibasic solution
HPS: Hydrogen peroxide solution
FE-SEM: Field emission scanning electron microscope
EDS: Energy-dispersive X-ray spectroscopy
wt%: Weight%
2D: Two-dimensional
3D: Three-dimensional
